# Comparative genomic analysis of mollicutes with and without a chaperonin system

**DOI:** 10.1371/journal.pone.0192619

**Published:** 2018-02-13

**Authors:** Dominik Schwarz, Orit Adato, Amnon Horovitz, Ron Unger

**Affiliations:** 1 University of Heidelberg, Faculty of Biosciences, Heidelberg, Germany; 2 The Mina Everard Goodman Faculty of Life Sciences, Bar-Ilan University, Ramat-Gan, Israel; 3 Department of Structural Biology, Weizmann Institute of Science, Rehovot, Israel; Academia Sinica, TAIWAN

## Abstract

The GroE chaperonin system, which comprises GroEL and GroES, assists protein folding *in vivo* and *in vitro*. It is conserved in all prokaryotes except in most, but not all, members of the class of mollicutes. In *Escherichia coli*, about 60 proteins were found to be obligatory clients of the GroE system. Here, we describe the properties of the homologs of these GroE clients in mollicutes and the evolution of chaperonins in this class of bacteria. Comparing the properties of these homologs in mollicutes with and without chaperonins enabled us to search for features correlated with the presence of GroE. Interestingly, no sequence-based features of proteins such as average length, amino acid composition and predicted folding/disorder propensity were found to be affected by the absence of GroE. Other properties such as genome size and number of proteins were also found to not differ between mollicute species with and without GroE. Our data suggest that two clades of mollicutes re-acquired the GroE system, thereby supporting the view that gaining the system occurred polyphyletically and not monophyletically, as previously debated. Our data also suggest that there might have been three isolated cases of lateral gene transfer from specific bacterial sources. Taken together, our data indicate that loss of GroE does not involve crossing a high evolutionary barrier and can be compensated for by a small number of changes within the few dozen client proteins.

## Introduction

Proteins can fold into their three-dimensional native structures spontaneously and without any assistance by other factors [1]. *In vivo*, however, protein aggregation and mis-folding can occur owing to macromolecular crowding and other conditions that exist in cells. Protein mis-folding is harmful to cells because of the costs involved in the synthesis and degradation of non-functional proteins, toxic effects of protein aggregates (such as disruption of cell membranes [2]) and the absence of functional protein molecules that may have crucial roles. Hence, it is not surprising that selection against mis-folding is a major driving force in evolution [3]. Molecular chaperones, which prevent aggregation and promote efficient protein folding, have, therefore, evolved and are found in all living cells [4,5]. The chaperone machineries that are involved in promoting protein substrate folding include the Hsp70 and Hsp90 systems and the chaperonins (Hsp60). These chaperones recognize regions that are exposed in non-native states of substrate proteins and they promote their folding by undergoing ATP-controlled cycles of protein substrate binding and release [6].

The chaperonins are a ubiquitous family of molecular chaperones that includes the GroE system from *Escherichia coli*. It comprises GroEL, an assembly formed by two rings of seven identical subunits, and GroES, which is a heptameric single-ring. ATP-dependent binding of GroES to one or both ends of GroEL results in formation of a cage(s) in which non-native proteins can be encapsulated, thereby preventing their aggregation [7] and, perhaps, also accelerating their folding [8].

Although GroEL can assist the folding *in vitro* of a wide range of proteins [9], it has become clear from theoretical considerations [10] and experimental studies [11] that it interacts *in vivo* with only about 250 proteins (out of a total number of about 4,300 proteins in *E*. *coli*). These proteins were partitioned by Kerner et al. [11] into three classes: 38 class I substrates that can be assisted by GroE but are also able to fold spontaneously; 126 class II substrates that require the GroE system at 37 °C but not at 25 °C; and 84 class III substrates that were found to be obligatory clients. A re-evaluation of class III proteins [12] led to defining a class IV that includes class III proteins that were verified to be stringent substrates and a small number of other proteins. Taken together, these experiments demonstrated that there is a core set of 57 *E*. *coli* proteins that are confirmed obligate substrates of the GroE system, i.e. proteins that will not fold *in vivo* or *in vitro* at 25 or 37 °C without GroE.

Mollicutes are a class of bacteria that lack a cell wall and are among the self-replicating organisms with the smallest genomes [13]. They are of special interest since members of this class are the only known organisms that lack a chaperonin system. The evolutionary track that led to the disappearance of the GroE system in most mollicute species and to its reappearance in some is not known but it is clear that there are closely related mollicute species that differ in whether they do or do not contain a chaperonin system.

Previously, Clark and Tillier [14] concluded that mollicutes did not evolve a protein that can functionally substitute for the GroE system. Hence, it was of interest to determine how mollicute homologs of the obligatory GroE clients in *E*. *coli* are able to fold in mollicute species that lack GroE. Towards this end, we compared mollicute species with a GroE homolog (GroE^+^) and those without one (GroE^-^). We then compared various properties of the homologs of the *E*. *coli* obligate GroE substrates (clients) and the *E*. *coli* control proteins (non-clients) in these two groups in order to determine whether, for example, certain types of sequence changes occurred that were able to compensate for the absence of a chaperonin system. This question has also been examined in previous more limited studies [14,15] that focused on a smaller number of mollicute species and fewer properties. Here, a larger set (59) of mollicute species is analyzed and more properties are compared. We also studied the evolution of the chaperonin system in mollicutes and re-examined whether the loss of a GroEL homolog occurred monophyletically [15] or polyphyletically [14]. Our findings support the latter view.

## Materials & methods

### Genome data collection

The *E*. *coli* genome was downloaded from NCBI: >gi|556503834|ref|NC_000913.3| *Escherichia coli* str. K-12 substr. MG1655 complete genome. The sequence of *E*. *coli* proteins was downloaded from UniProt using the following search terms: organism: “Escherichia coli (strain K12) [83333]" AND proteome: up000000625. The chosen reference genome of *E*. *coli* is 4.64 Mbp long and contains 4,306 proteins. The complete mollicute genomes were selected by NCBI Genome search: http://www.ncbi.nlm.nih.gov/genome/browse/. Resulting entries were downloaded into a text file containing clade ID, genome size and FTP links. A python script was used to select one genome per species, where species was defined by the clade ID. Some genomes lacked a clade ID and were, therefore, assigned the respective identifiers: ‘*Mycoplasma suis*’, ‘*Mycoplasma haemolamae*’, ‘*Mycoplasma arginine*’, ‘*Mycoplasma ovis*’, ‘*Mycoplasma wenyonii*’ and ‘*Mycoplasma parvum*’. Genomes with similar names to species with existing clade IDs were assigned those IDs: ‘*Mycoplasma canis*’: 21069, ‘*Mycoplasma mycoides*’: 21078, *‘Mycoplasma pneumoniae*': 21053 and ‘*Spiroplasma turonicum*': 39903'. The longest genome of each species was selected and downloaded from the NCBI ftp site. A local DNA BLAST database was then created for each of these genomes. In order to get the corresponding DNA sequences of the protein sequences, we downloaded the nucleotide sequences corresponding to the CDS annotation (*_cds_from_genomic.fna.gz) from ftp://ftp.ncbi.nlm.nih.gov/genomes/refseq/bacteria/ (These files are part of the data files provided for every assembly). The nucleotide sequences of 56 out of 59 mollicute species were downloaded since for 3 genomes the CDS are not available. The SILVA database [16] was used to retrieve 16S rRNA sequences (SSU) of the mollicute species.

### Comparing properties of proteins

For length, charge and FoldIndex comparisons, the mean of all homologs found in GroE^+^ or GroE^-^ of one query protein was calculated and then all (57 or less) the means (per GroE^+^ or GroE^-^ group) were averaged again for the group of clients or non-clients. For amino acid composition, amino acid substitution events and codon bias analysis, there is no meaning to “averaging”. Hence, every homolog (not every group of homologs per query protein) contributed equally.

### Codon bias analysis

In order to check whether the codon usage of the homologs of *E*. *coli* proteins differs between GroE^+^ and GroE^-^ mollicutes, the gi number of every protein was mapped to its Refseq ID via Uniport ID mapping. For the homologs of the *E*. *coli* GroE client proteins in mollicutes, 385 gi numbers (out of 454) were successfully mapped to their corresponding Refseq ID and for the homologs of the *E*. *coli* GroE non-client proteins in mollicutes, 165 gi numbers (out of 191) were successfully mapped to their corresponding Refseq ID. The DNA sequences of the homologs of the *E*. *coli* client and non-client proteins were downloaded from NCBI. Four Fasta files were created for the homologs of the *E*. *coli* clients and non-clients in GroE^+^ and GroE^-^ mollicutes, respectively. EMBOSS ([17]) CUSP was run on every file to analyze the codon usage. The output included fractions of codon usage for every DNA sequence that were added up to reflect the codon bias of the corresponding groups of proteins. The Shannon entropy of the codon usage for each amino acid (plus the Stop codons) was calculated according to $H\left(AA\right)=-\sum_{i=1}^{\#isocodons}P\left(codon\right)*log_{2}P\left(codon\right)$. Since Met and Trp residues are encoded by a single codon, their entropy is 0. Thus, comparison of entropies can be done for 19 (18 amino acids + stop) cases.

### Statistical analysis

The Welch's unequal variances t-test [18] was performed in order to determine whether various properties of sequences from GroE^+^ and GroE^-^ mollicute species are significantly different. The Welch’s t-test is a two-sample location test, which assumes normal distribution of populations without the additional assumption of equal variances and it tests if two populations have the same mean.

### Evolutionary tree construction

Phylogenetic and molecular evolution analysis of the GroE^+^ and GroE^-^ mollicute species listed in S1 and S2 Tables, were conducted using MEGA version 6 [19]. In the analysis, sequences were aligned using ClustalW and phylogenetic trees were constructed using the Maximum Likelihood method (and the MEGA6 default parameters) in accordance with the General Time Reversible and Gamma evolutionary models.

## Results

**Fig 1:**
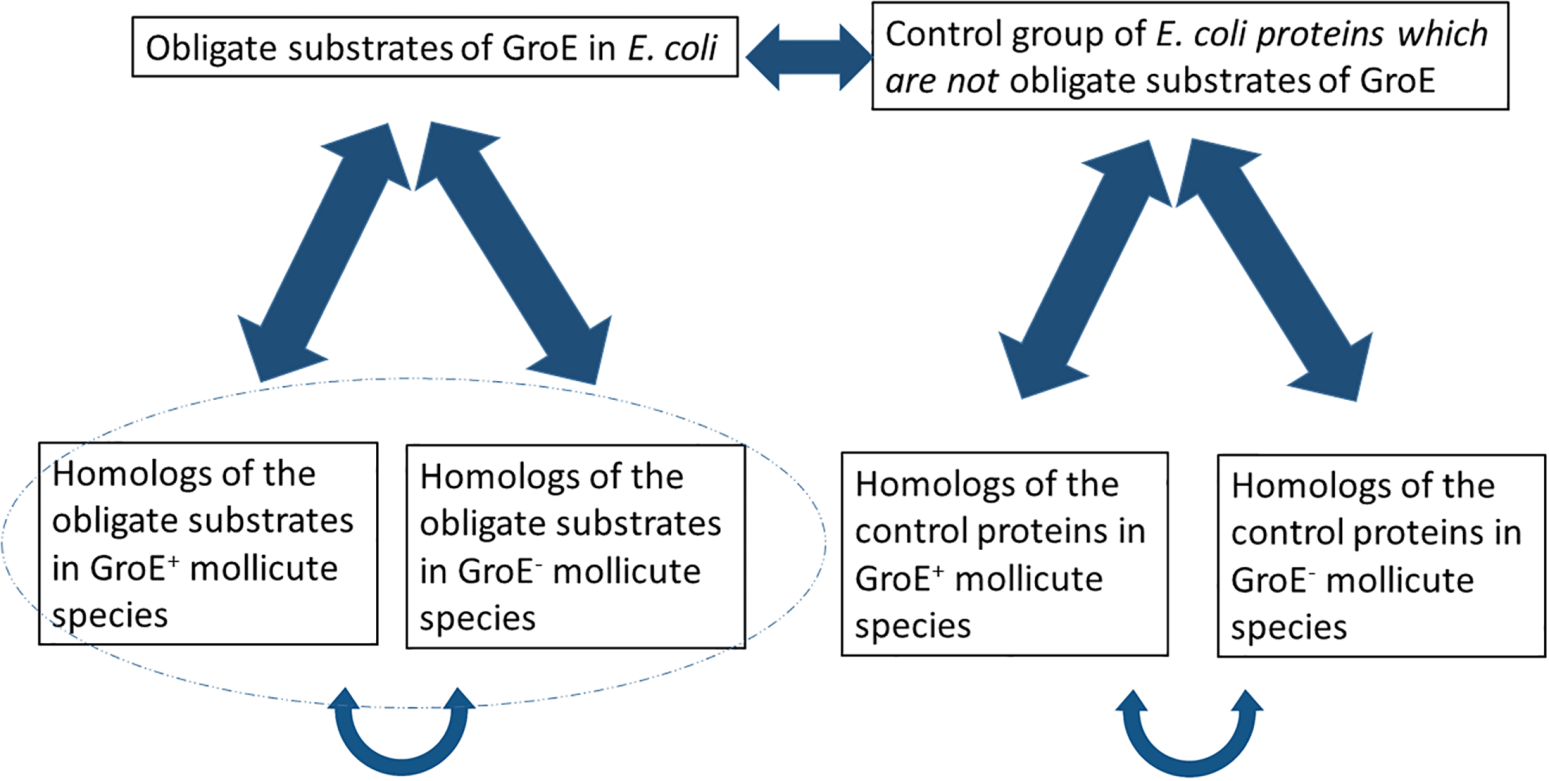
A scheme of the different groups of proteins used in this study. The ellipse shows the groups on which this work focuses but some properties are compared across all groups.

Many different comparisons can be made between *E*. *coli* clients and non-clients and their homologs in GroE^+^ and GroE^-^ mollicute species (Fig 1). For example, *E*. *coli* clients can be compared with their homologs in GroE^-^ mollicute species in order to uncover changes that occurred to compensate for the absence of GroE. Given, however, that *E*. *coli* and mollicutes are evolutionary distant and, thus, differ in many respects (e.g. GC content, codon usage, genome size etc.), a more controlled comparison is between the GroE^+^ and GroE^-^ mollicute homologs. Thus, most of the analysis here was done in this manner but, for completeness, we also compared other groups in Fig 1 with regard to some properties.

### General comparison of mollicute genomes with and without chaperonin systems

A search for homologs of *E*. *coli* GroEL (amino acid sequence P0A6F5) in the entire genomes of 59 mollicute species was carried out using TBLASTN with default parameters and an E-value threshold of 0.001. E-values of about 1e-100 were obtained for 13 species. We note that GroES was also present in all these species. Thus, these species were classified as GroE positive (GroE^+^). The other 46 species were classified as GroE negative (GroE^-^). The members of the GroE^+^ and GroE^-^ groups of species are listed in S1 and S2 Tables, respectively. The genome sizes of GroE^+^ and GroE^-^ species were then compared. The average genome sizes of GroE^+^ and GroE^-^ mollicutes were found to be 1.13 ± 0.40 and 0.95 ± 0.25 Mbp, respectively. This difference is, however, not statistically significant as indicated by a P-value of 0.152 obtained from a Welch's unequal variances t-test. The average number of proteins in GroE^+^ and GroE^-^ mollicutes was found to be 1022.23 ± 405.28 and 828.09 ± 279.64, respectively. This difference is also not statistically significant as indicated by a P-value of 0.138 obtained using the Welch's unequal variances t-test. The average lengths of proteins in the GroE^+^ and GroE^-^ mollicute species were found to be 315.35 ± 48.41 and 342.59 ± 48.41 amino acids, respectively. A P-value of 0.131 obtained using the Welch's unequal variances t-test indicated that also this difference is not significant.

### Identifying homologs of *E*. *coli* GroEL clients in mollicutes

A subset of class III clients identified by Kerner et al. [11] together with 4 obligate GroEL clients identified by Fujiwara et al. [12] were defined as class IV GroEL clients that comprises 57 proteins. A control set was created by selecting at random 57 *E*. *coli* proteins that are not GroEL clients and have the same length distribution as the clients (i.e. each client protein has a corresponding control protein with the same length except for 3 protein pairs that differ in their length by one amino acid). The length distributions were chosen to be the same since it is well established [20,21] that protein length is strongly correlated with folding properties. A search for homologs of the 57 obligate substrates and 57 control proteins in the 59 species of mollicutes was then carried out using BLASTP. Hits with an E-value threshold of 0.001 for which the sequences covered at least 80% of each other were considered as homologs. The lists of obligate substrates and control proteins are given in S3 and S4 Tables, respectively. In total, 130 and 340 homologs of the *E*. *coli* obligate clients were found in the GroE^+^ and GroE^-^ mollicute species, respectively. For the control group of *E*. *coli* non-clients, 53 and 152 homologs were found in the GroE^+^ and GroE^-^ mollicute species, respectively. The probability of finding a homolog of a client was higher than that of finding a homolog of a non-client control protein in both GroE^+^ and GroE^-^ mollicutes. For 28 *E*. *coli* obligate clients, at least one homolog was assigned in a GroE^+^ mollicute species and for 31 at least one in a GroE^-^ species. For 16 *E*. *coli* non-clients in the control group, at least one homolog was assigned in a GroE^+^ mollicute and for 19 at least one in a GroE^-^ species. For the number of homologs of each query protein, see the last two columns of S3 and S4 Tables.

### Comparing properties of client homologs in GroE^+^ and GroE^-^ mollicutes

The lengths of the homologs in GroE^+^ and GroE^-^ mollicutes of the obligate clients were compared (Table 1A) and the average length of the latter group was found to be slightly smaller. The difference is, however, not statistically significant. We also note that, in general, mollicute proteins tend to be shorter than their *E*. *coli* counterparts but also this difference is not statistically significant.

**Table 1:** Comparing properties of GroE^+^ and GroE^-^ homologs.

	Homologs in GroE^+^ mollicutes	^a^*E*. *coli*	Homologs in GroE^-^ mollicutes	^b^*E*. *coli*	^c^H0: is the mean equally distributed?
**A. Length**					
Clients	355.9 ± 72.8	362.3 ± 78.6	344.9 ± 69.9	352.3 ± 78.3	P-value: 0.563
Controls	331.9 ± 53.3	334.9 ± 65.7	343.0 ± 58.9	348.0 ± 61.5	P-value: 0.574
**B. Net charge**					
Clients	0.014 ± 0.009	0.025 ± 0.014	0.017 ± 0.007	0.024 ± 0.014	P-value: 0.192
Controls	0.017 ± 0.011	0.017 ± 0.010	0.019 ± 0.021	0.022 ± 0.014	P-value: 0.781
**C. FoldIndex**					
Clients	0.170 ± 0.041	0.171 ± 0.051	0.171 ± 0.041	0.179 ± 0.047	P-value: 0.980
Controls	0.203 ± 0.098	0.220 ± 0.092	0.196 ± 0.092	0.217 ± 0.081	P-value: 0.841

^a,b^ The values for the corresponding *E*. *coli* proteins (clients and control). Note that if an *E*. *coli* protein does not have even a single homolog in the relevant mollicute group then it was not included in the calculation. Thus, we get slightly different values for *E*. *coli* in the two columns.

^c^ The statistical significance between the GroE^+^ and GroE^-^ values.

A second feature we examined is protein net charge (Table 1B), which was defined as the absolute difference in the number of positively charged and negatively charged amino acid residues (at pH 7) divided by the total number of residues [22]. No statistically significant differences were found between the average net charges of the various groups. In a previous study [23] we noticed that GroE clients in *E*. *coli* have, on average, a lower folding propensity (as calculated by the FoldIndex program [24]) than random sets of *E*. *coli* proteins whereas control sets of proteins in *U*. *urealyticum* (which is a GroE^-^ organism) have a low FoldIndex. The current larger study shows (Table 1C) that mollicute proteins do have lower FoldIndex values than their *E*. *coli* counterparts, but this property is common to all mollicutes and is not unique to GroE^-^ organisms (i.e. the average FoldIndex values of proteins from GroE^+^ and GroE^-^ mollicutes are quite similar). Interestingly, however, the homologs of the *E*. *coli* GroE obligatory clients have a lower FoldIndex value, on average, than the homologs of the non-clients in both GroE^-^ and GroE^+^ species (Table 1).

The overall sequence compositions of proteins in *E*. *coli* and the GroE^+^ and GroE^-^ mollicutes are given in Table 2. Also given there are the sequence compositions of the GroE clients and control non-clients in *E*. *coli* and the homologs of these two groups in GroE^+^ and GroE^-^ mollicutes. There are clear differences in the sequence compositions of *E*. *coli* and the proteins of the mollicutes. Ala, Trp, Cys and Arg are strongly depleted in mollicute proteins while Ile, Asn and Lys are significantly enriched in mollicute proteins compared with their *E*. *coli* homologs. Interestingly, Lys residues in mollicutes are about 3 times more frequent than Arg whereas in *E*. *coli* the frequency of Lys is slightly smaller than that of Arg. No significant differences are observed, however, between the protein sequence composition of GroE^+^ and GroE^-^ mollicutes. There are also no significant differences between the compositions of the homologs of the GroE client and non-clients in the protein sequences of the GroE^+^ and GroE^-^ mollicutes.

**Table 2:** Amino acid composition [%] of the GroEL clients and non-clients in E. coli and their homologs in GroE^+^ and GroE^-^ mollicutes.

**GroEL positive mollicutes**:																				
	**A**	**V**	**M**	**L**	**I**	**P**	**W**	**F**	**Y**	**T**	**Q**	**G**	**S**	**C**	**N**	**H**	**K**	**R**	**E**	**D**
Proteome	5.06	5.90	2.06	10.03	9.14	2.89	0.83	5.31	4.36	5.64	3.83	5.02	6.39	0.67	7.09	1.65	9.45	3.02	6.31	5.34
Class IV client homologs	6.84	6.54	2.22	9.23	8.83	3.12	0.57	4.48	3.62	5.13	3.40	6.92	5.88	0.98	6.44	2.29	9.14	2.43	6.47	5.48
Nonclient homologs	6.62	7.34	2.09	10.05	9.67	3.51	0.53	3.95	3.52	5.30	3.73	5.72	5.81	0.71	5.66	2.02	9.59	2.85	5.90	5.42
**GroEL negative mollicutes**:																				
	**A**	**V**	**M**	**L**	**I**	**P**	**W**	**F**	**Y**	**T**	**Q**	**G**	**S**	**C**	**N**	**H**	**K**	**R**	**E**	**D**
Proteome	5.11	5.57	1.84	9.63	9.31	2.71	1.06	5.42	4.03	5.16	3.36	5.02	7.00	0.69	7.38	1.41	10.06	2.99	6.84	5.42
Class IV client homologs	6.90	6.26	2.15	8.99	9.62	3.12	0.72	4.42	3.56	4.79	2.88	6.60	6.07	0.94	6.34	1.89	9.54	2.61	6.95	5.73
Nonclient homologs	6.91	7.23	1.77	9.27	9.64	3.22	0.64	3.99	3.21	5.42	3.10	6.28	6.14	0.83	6.03	1.81	9.50	2.85	6.34	5.82
**E. coli**																				
	**A**	**V**	**M**	**L**	**I**	**P**	**W**	**F**	**Y**	**T**	**Q**	**G**	**S**	**C**	**N**	**H**	**K**	**R**	**E**	**D**
Proteome	9.52	7.07	2.82	10.67	6.01	4.43	1.53	3.89	2.85	5.40	4.44	7.37	5.80	1.16	3.95	2.27	4.41	5.51	5.76	5.15
Class IV clients	10.08	6.84	3.02	9.69	5.51	4.50	1.28	3.68	2.88	5.15	4.13	8.04	5.16	1.32	3.70	2.85	4.15	5.95	6.15	5.92
Nonclients	10.51	7.19	3.09	10.63	6.18	4.52	1.67	3.87	2.81	5.34	4.15	7.86	5.56	1.22	3.77	2.33	4.35	4.61	5.36	4.96

The overall compositions of *E*. *coli* and the GroE^+^ and GroE^-^ mollicutes are also provided. The blue cells mark amino acid for which the frequency in mollicute is higher (color gradient starting with 10% difference) compared to the corresponding amino acid frequency in *E*. *coli*, while the red cells mark amino acid with frequency that is lower (color gradient starting with 10% difference) compared with the corresponding frequency in *E*. *coli*.

### Multiple sequence alignments

As mentioned above, for the 57 client sequences, 28 and 31 sets of homologs were found in the GroE^+^ and GroE^-^ mollicute species, respectively, and for the non-clients, 16 and 19 set of homologs were found in the GroE^+^ and GroE^-^ mollicute species, respectively. The sequences of the 57 *E*. *coli* obligate GroEL clients and the 57 control non-clients were aligned using MUSCLE with their respective homologs. The heat maps in S1 and S2 Figs show the rate of substitution of each amino acid between the *E*. *coli* clients (on the vertical axis) and the aligned positions in GroE^+^ and GroE^-^ mollicute homologs (on the horizontal axis). The rates were calculated by counting the number of changes at all positions in the mollicute sequences and then normalizing to 1. While there are differences in the values within each matrix and between the matrices, we could not identify any large changes or consistent patterns in these matrices.

### Codon usage

We compared the codon usage of the homologs of the *E*. *coli* clients in GroE^+^ and GroE^-^ mollicutes. S5 Table (left columns) shows the fraction of usage of every codon for each amino acid in the client homologs in GroE^+^ and GroE^-^ mollicutes. Overall, the codon usage is quite similar but there are several differences that can be noticed. First, there are noticeable differences in the usage of TAA (stop) and GGA (Gly) codons. Moreover and interestingly, the entropies of the codon usage for 17 out of the 19 cases are lower for the client homologs in GroE^+^ mollicutes than in the GroE^-^ mollicutes. However, these differences are not unique to the client homologs. Similar results are found (S5 Table, right columns) when comparing the control (i.e. the non-clients) codon usage. Again, the main difference seems to be in the usage of the TAA and GGA codons between the GroE^+^ and GroE^-^ mollicute species. Also, when comparing the entropies, we noticed that for 16 out of the 19 amino acids the entropy is lower in the case of the non-client homologs in GroE^+^ mollicutes than in the GroE^-^ mollicutes. This difference in the entropies between GroE^+^ and GroE^-^ mollicutes is intriguing but it is not a property that distinguishes between the client homologs in GroE^+^ and GroE^-^ mollicutes.

### The evolution of the GroE system in mollicutes

**Fig 2:**
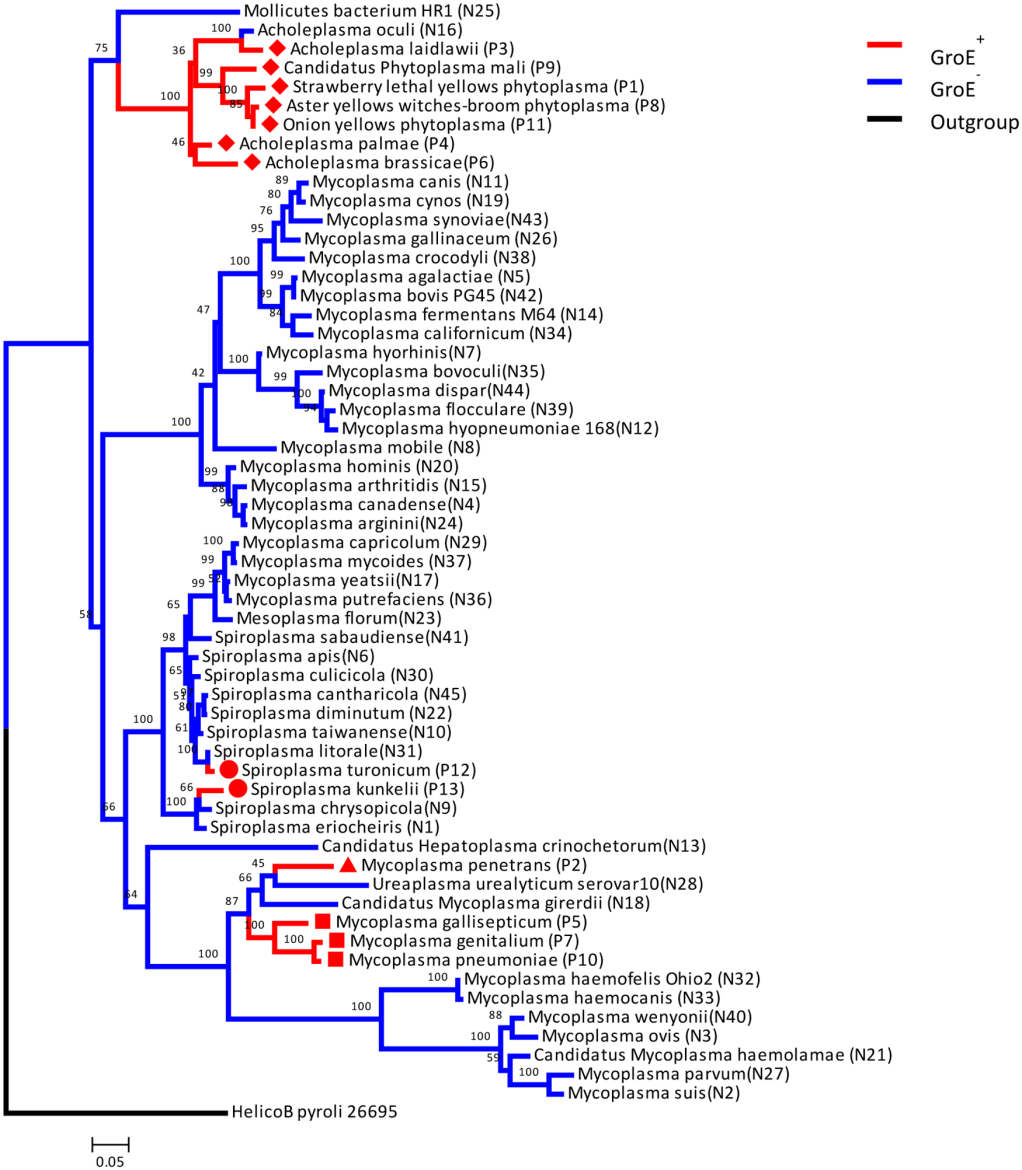
Distribution of the GroE system in a 16S rRNA-based evolution tree of mollicutes. The branches of the GroE^+^ and GroE^-^ species are marked in red and blue, respectively. The numbers on the branching points represent the bootstrapping frequencies, the branch length represents the sequence distance and the scale shows a distance of 5%. The diamonds and squares show the two major clades that contain the GroE system. The triangle marks the species where lateral gene transfer (LGT) was suggested before and circles mark the two additional species for which we suggest LGT events.

The evolutionary tree of the distribution of the GroE system in mollicutes was constructed as described in the Methods (Fig 2). Mollicutes have evolved from other bacteria and underwent reductive evolution and lost many of their genes [25]. Given that the GroE system is absent in most branches of the evolutionary tree of this class, it is likely that the root of the mollicute tree lost the GroE system and that it re-emerged polyphyletically in several branches of the tree. We can distinguish two major clades that contain the GroE system (Fig 2): a big clade that includes seven species, acholeplasma and phytoplasma, marked with red diamonds (P3, P9, P1, P8, P11, P4 & P6), and a smaller clade that includes three species marked with red squares (P5, P7, P10).

**Fig 3:**
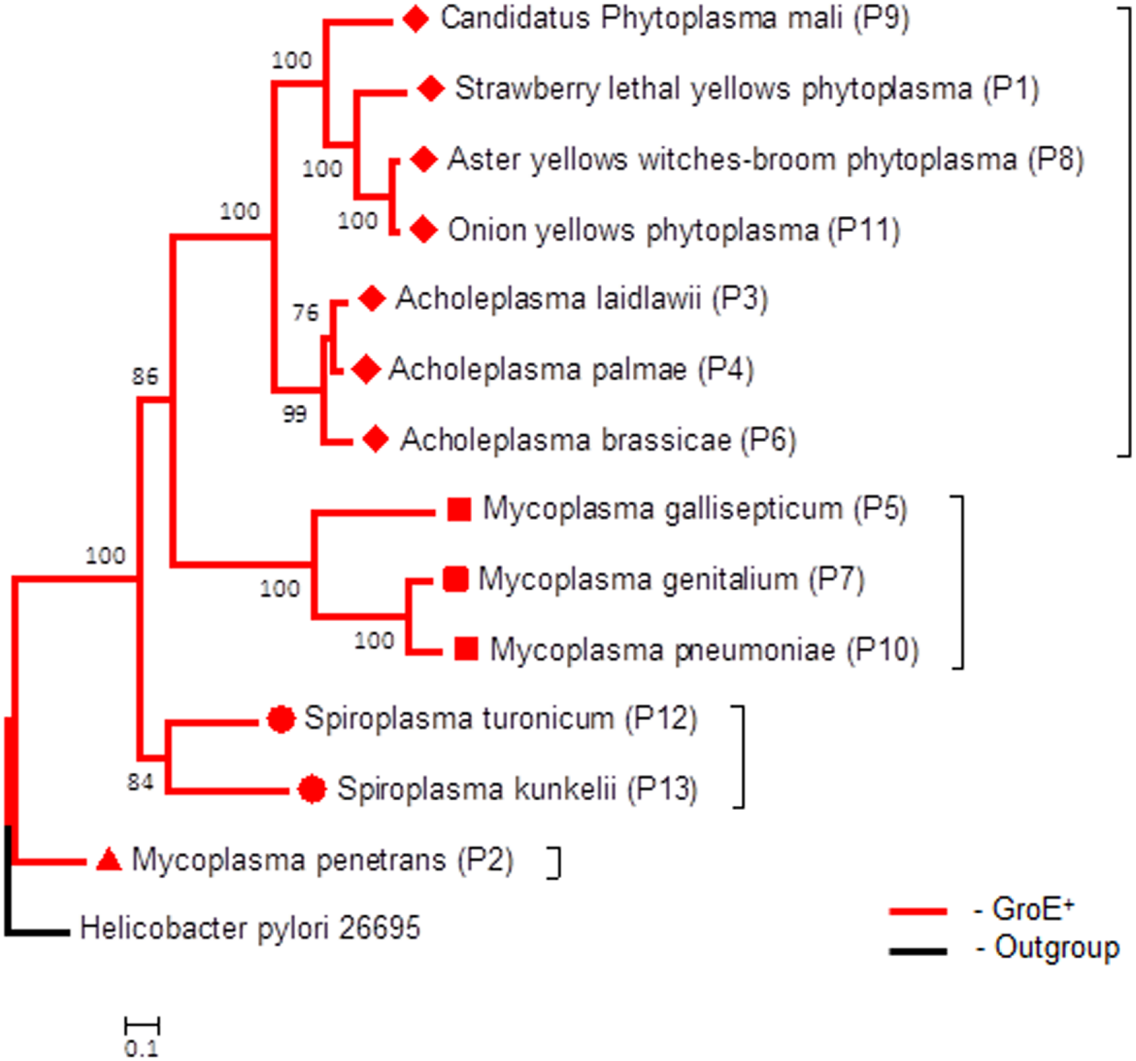
Evolutionary tree of GroEL sequences of GroE^+^ mollicutes. The diamonds and squares show the two major clades that contain the GroE system. The triangle marks the species where lateral gene transfer (LGT) was suggested before and circles mark the two additional species for which we suggest LGT events.

Of special interest are the two species *Spiroplasma kunkelii* (P13) and *Spiroplasma turonicum* (P12) (marked with red circles in Figs 2 and 3) that have the GroE system whereas their immediate evolutionary neighbors do not. Interestingly, their GroEL sequences and GroES sequences show high similarity to the GroEL and GroES sequences from bacteria belonging to the *Bacilli* class. BlastP shows that GroEL from *Spiroplasma turonicum* (P12) has 85% sequence similarity (71% identity) to GroEL from *Enterococcus raffinosus* with a 96% query coverage. GroEL from *Spiroplasma kunkelii* (P13) has 80% sequence similarity (65% identity) to that from *Enterococcus raffinosus* with a 96% query coverage. The sequence similarity between GroELs from *Spiroplasma turonicum* and *Spiroplasma kunkelii* is 78%. The similarity between these two GroE^+^ spiroplasma to the GroEL of the closest other mollicutes is lower and is about 75 and 71%, respectively. GroES from *Spiroplasma turonicum* (P12) has 62% sequence similarity (43% identity) to GroES from *Salinicoccus sediminis* with 97% query coverage. GroES from *Spiroplasma kunkelii* (P13) has 64% sequence similarity (43% identity) to GroES from *Gorillibacterium massiliense* with 98% query coverage. These results point to a possible event of lateral gene transfer between one of the species from the Bacilli class and *S*. *kunkelii* (P13) and *S*. *turonicum* (P12). Our analysis also points to a lateral gene transfer event for the emergence of the GroE system in *Mycoplasma penetrans* (P2, marked with a red triangle) as both the GroEL and GroES sequences from *Mycoplasma penetrans* show higher similarity to their homologues in *Helicobacter pylori* than to those in any mollicutes species. The GroEL from *mycoplasma penetrans* (P2) has 85% sequence similarity and 70% sequence identity to GroEL from *Helicobacter pylori* with 98% coverage, while the GroES sequence from *Mycoplasma penetrans* (P2) has 71% sequence similarity (43% identity) with 97% query coverage to GroES from *Helicobacter pylor*i. This finding confirmed the lateral gene transfer previously observed by Clark and Tillier [14]. Building the same tree with neighbor joining using the PHYLIP software resulted in the same branch structure (data not shown). In addition to the tree above, we built separate evolutionary trees for GroEL and GroES sequences for the GroEL^+^ organisms. Both trees of GroEL and GroES sequences coincide with the “standard” evolutionary tree presented in Fig 2, which is based on 16S rRNA with minor differences. The GroEL tree is presented in Fig 3 and the similar GroES tree is presented in S3 Fig (*Helicobacter pyroli* 26695 was used as an outgroup in the evolutionary trees in Fig 3 and S3 Fig).

## Discussion

Our analysis shows that 13 species of mollicutes are GroE^+^ and 46 are GroE^-^. Previous experimental studies [26,27] reported the existence of a GroEL homolog in *Mycoplasma fermentans* and *Mycoplasma suis* but we did not detect one in these species and, thus, classified them as GroE^-^ (the existence of the GroE system in *Mycoplasma suis* has also been challenged before [28].

In all aspects that we checked (i.e. genome size, number of proteins, average protein length, amino acid composition, folding propensities and codon bias), no statistically significant differences are observed between homologs of *E*. *coli* GroE clients in GroE^+^ and GroE^-^ mollicute species. Our data do not support, therefore, the suggestion [29] that an excess of Lys and Arg (when compared to the *E*. *coli* proteins) is related to the ability of N-acetylneuraminic acid aldolase from *M*. *synoviae*, a GroE^-^ organism, to fold in the absence of GroE. Our data show (Table 1) that the excess of Lys at the expense of Arg is, in fact, a general property of mollicutes that does not depend on whether they do or do not contain the GroE system. Similarly, the suggestion by Georgescauld et al. [29] that the number of Phe and Tyr residues is higher in the GroE^-^ species compared to the GroE^+^ species is not supported by our data, which show that this is a general property of mollicutes regardless of their GroE content.

Obvious differences between *E*. *coli* proteins that are obligate clients of the GroE systems and those that are not have not been identified so far [30]. Nevertheless, the loss of the GroE system in the root of the mollicute class may have conferred a strong and immediate pressure on proteins that originate from obligatory clients to escape from the GroE system. According to this evolutionary scenario, systematic changes in the sequences of these proteins might have been expected. Our study suggests, however, that no large systematic differences exist between the sequence features of proteins that originated from clients of the GroE system and those that are not dependent on the GroE system. This observation is consistent with the report [31] that single amino acid changes are sufficient to convert a GroE-independent protein into a dependent one. Furthermore, in a recent study [32] we studied the GroEL dependence of GFP, a eukaryotic protein that is often used as a fluorescent marker also in prokaryotic systems and folds in a GroE-dependent manner. We found that single mutations in GFP can decrease the GroEL dependence of its folding. These mutations were of residues at “frustrated” positions [33]. The conclusion of that study is that even a single mutation is sufficient to change the GroE dependence of a protein. This conclusion is consistent with the fact that large-scale differences between homologs of *E*. *coli* obligate substrate in GroE+ and GroE- organisms were not found in this study and in two previous studies [13,14]. It is also in accord with our conclusion (Fig 2) that regaining the GroE system occurred in a polyphyletic manner [14] and not monophyletically as suggested before [15]. The fact that we have many closely related species with and without the GroE system suggests that compensation for the absence of the GroE system does not require crossing a high evolutionary barrier and can be achieved, as argued above, by a small number of changes within the few dozen proteins that are dependent on GroE in other organisms.

Given such a compensatory mechanism, one can ask why there are several mollicute species that have regained the GroE system. We note that GroEL has been suggested to be a moonlighting protein, i.e. to have roles other than in assisted folding [34] of which some may be related to pathogenicity (see [35]). Thus, it is possible that the regain of GroEL in some mollicute species was driven by functions of GroEL not related to its role in folding.

## Supporting information

S1 Table Mollicute species with a GroEL homolog in their genome (GroE^+^). (DOCX)

S2 Table Mollicute species with no GroEL homolog in their genome (GroE^-^). (DOCX)

S3 Table List of *E*. *coli* obligate GroEL clients. (DOCX)

S4 Table List of selected *E*. *coli* GroEL non-clients (control). (DOCX)

S5 Table Analysis of codon usage and codon usage entropies. (DOCX)

S1 Fig Amino acid substitution events from *E*. *coli* class IV clients to their homologs [%]. The upper matrix shows substitution events to homologs found in GroE^+^ mollicutes. The lower matrix shows substitutions in homologs found in GroE^-^ mollicute species.
(DOCX)

S2 Fig Amino acid substitution events from *E*. *coli* non-clients to their homologs [%]. The upper matrix displays substitution events to homologs found in GroE^+^ mollicutes. The lower matrix shows substitutions in homologs found in GroE^-^ species.
(DOCX)

S3 Fig Evolutionary tree of GroES sequences of GroE^+^ mollicutes. The diamonds and squares show the two major clades that contain the GroE system. The triangle marks the species where lateral gene transfer (LGT) was suggested before and circles mark the two additional species for which we suggest LGT events.
(DOCX)
