# Supplementary material for: Comparative genomic analysis of mollicutes with and without a chaperonin system
Source: PLoS One. 2018 Feb 13;13(2):e0192619. doi: 10.1371/journal.pone.0192619 (PMC5810989; doi:10.1371/journal.pone.0192619)
Supplement: S1 Table — (DOCX) [file pone.0192619.s001.docx]

S1 Table. Mollicute species with a GroEL homolog in their genome (GroE^+^)

| Tag | *Species name* | E-value | Accession ID |
| --- | --- | --- | --- |
|  |  |  |  |
| P1 | *Strawberry lethal yellows phytoplasma (CPA)* str. NZSb11 | 1.00E-114 | CP002548.1 |
| P2 | *Mycoplasma penetrans* HF-2 DNA | 1.00E-143 | BA000026.2 |
| P3 | *Acholeplasma laidlawii* PG-8A | 1.00E-131 | CP000896.1 |
| P4 | *Complete chromosome Acholeplasma palmae* | 1.00E-130 | FO681347.1 |
| P5 | *Mycoplasma gallisepticum* str. R(low) | 1.00E-101 | AE015450.2 |
| P6 | *Complete chromosome Acholeplasma brassicae* | 1.00E-131 | FO681348.1 |
| P7 | *Mycoplasma genitalium* G37 | 3.00E-97 | L43967.2 |
| P8 | *Aster yellows witches'-broom phytoplasma* AYWB | 1.00E-113 | CP000061.1 |
| P9 | *Candidatus Phytoplasma mali* strain AT | 1.00E-114 | CU469464.1 |
| P10 | *Mycoplasma pneumoniae* M29 | 3.00E-99 | CP008895.1 |
| P11 | *Onion yellows phytoplasma* OY-M DNA | 1.00E-113 | AP006628.2 |
| P12 | *Spiroplasma turonicum* strain Tab4c | 1.00E-136 | CP012328.1 |
| P13 | *Spiroplasma kunkelii* CR2-3x | 1.00E-131 | CP010899.1 |
