# Supplementary material for: Comparative genomic analysis of mollicutes with and without a chaperonin system
Source: PLoS One. 2018 Feb 13;13(2):e0192619. doi: 10.1371/journal.pone.0192619 (PMC5810989; doi:10.1371/journal.pone.0192619)
Supplement: S2 Table — (DOCX) [file pone.0192619.s002.docx]

S2 Table. Mollicute species with no GroEL homolog in their genome (GroE^-^)

| Tag | Species name | Accession ID |
| --- | --- | --- |
| N1 | *Spiroplasma eriocheiris* CCTCC M 207170 | CP011856.1 |
| N2 | *Mycoplasma suis* str. Illinois | CP002525.1 |
| N3 | *Mycoplasma ovis* str. Michigan | CP006935.1 |
| N4 | *Mycoplasma canadense* HAZ360_1 DNA | AP014631.1 |
| N5 | *Mycoplasma agalactiae* 5632 chromosome | FP671138.1 |
| N6 | *Spiroplasma apis* B31 | CP006682.1 |
| N7 | *Mycoplasma hyorhinis* HUB-1 | CP002170.1 |
| N8 | *Mycoplasma mobile* 163K | AE017308.1 |
| N9 | *Spiroplasma chrysopicola* DF-1 | CP005077.1 |
| N10 | *Spiroplasma taiwanense* CT-1 | CP005074.1 |
| N11 | *Mycoplasma canis* strain LV | CP011368.1 |
| N12 | *Mycoplasma hyopneumoniae* 168 | CP002274.1 |
| N13 | Candidatus Hepatoplasma crinochetorum Av | CP006932.1 |
| N14 | Mycoplasma fermentans M64 | CP002458.1 |
| N15 | *Mycoplasma arthritidis* 158L3-1 | CP001047.1 |
| N16 | *Acholeplasma oculi strain* 19L | LK028559.1 |
| N17 | *Mycoplasma yeatsii* GM274B | CP007520.1 |
| N18 | *Candidatus Mycoplasma girerdii* strain VCU_M1 | CP007711.1 |
| N19 | *Mycoplasma cynos* C142 | HF559394.1 |
| N20 | *Mycoplasma hominis* ATCC 27545 | CP009652.1 |
| N21 | *Candidatus Mycoplasma haemolamae* str. Purdue | CP003731.1 |
| N22 | *Spiroplasma diminutum* CUAS-1 | CP005076.1 |
| N23 | *Mesoplasma florum* W37 | CP006778.1 |
| N24 | *Mycoplasma arginini* DNA | AP014657.1 |
| N25 | *Mollicutes bacterium* HR1 | CP009415.1 |
| N26 | *Mycoplasma gallinaceum* strain B2096 8B | CP011021.1 |
| N27 | *Mycoplasma parvum* str. Indiana | CP006771.1 |
| N28 | *Ureaplasma urealyticum serovar 10* str. ATCC 33699 | CP001184.1 |
| N29 | *Mycoplasma capricolum subsp. capripneumoniae* 87001 | CP006959.1 |
| N30 | *Spiroplasma culicicola* AES-1 | CP006681.1 |
| N31 | *Spiroplasma litorale strain* TN-1 | CP012357.1 |
| N32 | *Mycoplasma haemofelis* Ohio2 | CP002808.1 |
| N33 | *Mycoplasma haemocanis* str. Illinois | CP003199.1 |
| N34 | *Mycoplasma californicum* HAZ160_1 DNA | AP013353.1 |
| N35 | *Mycoplasma bovoculi* M165/69 | CP007154.1 |
| N36 | *Mycoplasma putrefaciens* Mput923*1* | CP004357.1 |
| N37 | *Mycoplasma mycoides subsp. mycoides* SC str. PG1 | BX293980.2 |
| N38 | *Mycoplasma crocodyli* MP145 | CP001991.1 |
| N39 | *Mycoplasma flocculare* ATCC 27399 | CP007585.1 |
| N40 | *Mycoplasma wenyonii* str. Massachusetts | CP003703.1 |
| N41 | *Spiroplasma sabaudiense* Ar-1343 | CP006934.1 |
| N42 | *Mycoplasma bovis* PG45 clone MU clone A2 | CP002188.1 |
| N43 | *Mycoplasma synoviae* ATCC 25204 | CP011096.1 |
| N44 | *Mycoplasma dispar strain* ATCC 27140 | CP007229.1 |
| N45 | *Spiroplasma cantharicola strain* CC-1 | CP012622.1 |
| N46* | *Mycoplasma sp. (ex Biomphalaria glabrata* | CP013128. |

* The 16S sequence of this species was not found in the SILVA database and, thus, was excluded from the evolutionary analysis.
