# Supplementary material for: Comparative genomic analysis of mollicutes with and without a chaperonin system
Source: PLoS One. 2018 Feb 13;13(2):e0192619. doi: 10.1371/journal.pone.0192619 (PMC5810989; doi:10.1371/journal.pone.0192619)
Supplement: S3 Table — (DOCX) [file pone.0192619.s003.docx]

**S3 Table. List of *E. coli* obligate GroEL clients**

| Entry | Entry name | Protein name | Gene name | Length | Homologs (GroE+) | Homologs (GroE-) |
| --- | --- | --- | --- | --- | --- | --- |
| P08202 | ARAA_ECOLI | L-arabinose isomerase  (EC 5.3.1.4) | araA | 500 | 0 | 0 |
| P0A8U2 | YAFD_ECOLI | UPF0294 protein YafD | yafD | 266 | 0 | 0 |
| P21515 | ACPH_ECOLI | Acyl carrier protein phosphodiesterase  (EC 3.1.4.14) | acpH | 193 | 0 | 0 |
| P77735 | YAJO_ECOLI | Uncharacterized oxidoreductase YajO  (EC 1.-.-.-) | yajO | 324 | 1 | 6 |
| P60716 | LIPA_ECOLI | Lipoyl synthase (EC 2.8.1.8) | lipA | 321 | 0 | 0 |
| P0AC41 | SDHA_ECOLI | Succinate dehydrogenase flavoprotein subunit  (EC 1.3.5.1) | sdhA | 588 | 0 | 0 |
| P12998 | BIOF_ECOLI | 8-amino-7-oxononanoate synthase (AONS)  (EC 2.3.1.47) | bioF | 384 | 0 | 3 |
| P75821 | YBJS_ECOLI | Uncharacterized protein YbjS | ybjS | 337 | 1 | 1 |
| P75823 | LTAE_ECOLI | Low specificity L-threonine aldolase (EC 4.1.2.48) | ltaE | 333 | 1 | 1 |
| P23721 | SERC_ECOLI | Phosphoserine aminotransferase  (EC 2.6.1.52) | serC | 362 | 0 | 0 |
| P0A7E1 | PYRD_ECOLI | Dihydroorotate dehydrogenase (quinone)  (EC 1.3.5.2) | pyrD | 336 | 2 | 4 |
| P05020 | PYRC_ECOLI | Dihydroorotase (DHOase) (EC 3.5.2.3) | pyrC | 348 | 0 | 0 |
| P0AAI5 | FABF_ECOLI | 3-oxoacyl-[acyl-carrier-protein] synthase 2  (EC 2.3.1.179) | fabF | 413 | 3 | 2 |
| P0AFQ7 | YCFH_ECOLI | Uncharacterized deoxyribonuclease YcfH  (EC 3.1.21.-) | ycfH | 265 | 13 | 34 |
| P75949 | NAGZ_ECOLI | Beta-hexosaminidase  (EC 3.2.1.52) | nagZ | 341 | 0 | 0 |
| P0A6J5 | DADA_ECOLI | D-amino acid dehydrogenase (EC 1.4.99.-) | dadA | 432 | 0 | 0 |
| P0A715 | KDSA_ECOLI | 2-dehydro-3-deoxyphosphooctonate aldolase (EC 2.5.1.55) | kdsA | 284 | 0 | 0 |
| P76143 | LSRF_ECOLI | Uncharacterized aldolase LsrF (EC 4.1.2.-) | lsrF | 291 | 0 | 0 |
| P38104 | RSPA_ECOLI | Starvation-sensing protein RspA | rspA | 404 | 1 | 1 |
| P22333 | ADD_ECOLI | Adenosine deaminase  (EC 3.5.4.4) | add | 333 | 0 | 1 |
| P77444 | SUFS_ECOLI | Cysteine desulfurase  (EC 2.8.1.7) | sufS | 406 | 13 | 37 |
| P00370 | DHE4_ECOLI | NADP-specific glutamate dehydrogenase (EC 1.4.1.4) | gdhA | 447 | 2 | 1 |
| P37745 | RMLC_ECOLI | dTDP-4-dehydrorhamnose 3,5-epimerase (EC 5.1.3.13) | rfbC | 185 | 0 | 0 |
| P0C8J6 | GATY_ECOLI | D-tagatose-1,6-bisphosphate aldolase subunit GatY  (EC 4.1.2.40) | gatY | 284 | 13 | 31 |
| P33371 | DUSC_ECOLI | tRNA-dihydrouridine(16) synthase (EC 1.3.1.-) | dusC | 315 | 0 | 0 |
| P33599 | NUOCD_ECOLI | NADH-quinone oxidoreductase subunit C/D (EC 1.6.5.11) | nuoC | 596 | 0 | 0 |
| P0A959 | ALAA_ECOLI | Glutamate-pyruvate aminotransferase AlaA  (EC 2.6.1.2) | alaA | 405 | 0 | 1 |
| P0AEJ6 | EUTB_ECOLI | Ethanolamine ammonia-lyase heavy chain (EC 4.3.1.7) | eutB | 453 | 0 | 0 |
| P0A6L2 | DAPA_ECOLI | 4-hydroxy-tetrahydrodipicolinate synthase (EC 4.3.3.7) | dapA | 292 | 2 | 2 |
| P77475 | YQAB_ECOLI | Fructose-1-phosphate phosphatase YqaB  (EC 3.1.3.-) | yqaB | 188 | 0 | 11 |
| P0ACK8 | FUCR_ECOLI | L-fucose operon activator | fucR | 243 | 4 | 5 |
| P0A8S1 | ARGP_ECOLI | HTH-type transcriptional regulator ArgP | argP | 297 | 0 | 0 |
| P0A817 | METK_ECOLI | S-adenosylmethionine synthase (AdoMet synthase) (EC 2.5.1.6) | metK | 384 | 9 | 44 |
| P0A8G3 | UXAC_ECOLI | Uronate isomerase  (EC 5.3.1.12) | uxaC | 470 | 2 | 2 |
| P0A6L4 | NANA_ECOLI | N-acetylneuraminate lyase (EC 4.1.3.3) | nanA | 297 | 1 | 5 |
| P0AGG8 | TLDD_ECOLI | Metalloprotease TldD  (EC 3.4.-.-) | tldD | 481 | 6 | 2 |
| P0ABT5 | DUSB_ECOLI | tRNA-dihydrouridine synthase B (EC 1.3.1.-) | dusB | 321 | 4 | 16 |
| P0A9R7 | FTSE_ECOLI | Cell division ATP-binding protein FtsE | ftsE | 222 | 4 | 9 |
| P00944 | XYLA_ECOLI | Xylose isomerase  (EC 5.3.1.5) | xylA | 440 | 1 | 0 |
| P37685 | ALDB_ECOLI | Aldehyde dehydrogenase B (EC 1.2.1.-) | aldB | 512 | 3 | 27 |
| P33232 | LLDD_ECOLI | L-lactate dehydrogenase  (EC 1.1.-.-) | lldD | 396 | 0 | 0 |
| P27859 | TATD_ECOLI | Tat-linked quality control protein TatD (EC 3.1.21.-) | tatD | 260 | 13 | 29 |
| P21151 | FADA_ECOLI | 3-ketoacyl-CoA thiolase  (EC 2.3.1.16) | fadA | 387 | 3 | 2 |
| P21165 | PEPQ_ECOLI | Xaa-Pro dipeptidase  (EC 3.4.13.9) | pepQ | 443 | 3 | 2 |
| P0AEZ1 | METF_ECOLI | 5,10-methylenetetrahydrofolate reductase (EC 1.5.1.20) | metF | 296 | 0 | 0 |
| P23908 | ARGE_ECOLI | Acetylornithine deacetylase (EC 3.5.1.16) | argE | 383 | 0 | 0 |
| P30140 | THIH_ECOLI | 2-iminoacetate synthase  (EC 4.1.99.19) | thiH | 377 | 0 | 0 |
| P00363 | FRDA_ECOLI | Fumarate reductase flavoprotein subunit  (EC 1.3.5.4) | frdA | 602 | 0 | 0 |
| P0AFK0 | PMBA_ECOLI | Metalloprotease PmbA  (EC 3.4.-.-) | pmbA | 450 | 5 | 0 |
| P39359 | YJHH_ECOLI | Uncharacterized lyase YjhH (EC 4.-.-.-) | yjhH | 301 | 1 | 2 |
| P39407 | YJJU_ECOLI | Uncharacterized protein YjjU (EC 3.1.1.-) | yjjU | 357 | 0 | 0 |
| P07650 | TYPH_ECOLI | Thymidine phosphorylase (EC 2.4.2.4) | deoA | 440 | 9 | 35 |
| P0ACB2 | HEM2_ECOLI | Delta-aminolevulinic acid dehydratase (EC 4.2.1.24) | hemB | 324 | 0 | 0 |
| P0A9Q9 | DHAS_ECOLI | Aspartate-semialdehyde dehydrogenase (EC 1.2.1.11) | asd | 367 | 1 | 1 |
| P0A6C1 | END4_ECOLI | Endonuclease 4 (EC 3.1.21.2) | nfo | 285 | 9 | 40 |
| P0A991 | ALF1_ECOLI | Fructose-bisphosphate aldolase class 1 (EC 4.1.2.13) | fbaB | 350 | 0 | 0 |
| P29012 | ALR2_ECOLI | Alanine racemase, catabolic (EC 5.1.1.1) | dadX | 356 | 0 | 1 |
