# Supplementary material for: Comparative genomic analysis of mollicutes with and without a chaperonin system
Source: PLoS One. 2018 Feb 13;13(2):e0192619. doi: 10.1371/journal.pone.0192619 (PMC5810989; doi:10.1371/journal.pone.0192619)
Supplement: S4 Table — (DOCX) [file pone.0192619.s004.docx]

S4 Table. List of selected *E. coli* GroEL non-clients (control)

| Entry | Entry name | Protein name | Gene name | Length | Homologs (GroE+) | Homologs (GroE-) |
| --- | --- | --- | --- | --- | --- | --- |
| P77304 | DTPA_ECOLI | Dipeptide and tripeptide permease A | dtpA | 500 | 0 | 0 |
| P37028 | BTUF_ECOLI | Vitamin B12-binding protein | btuF | 266 | 0 | 0 |
| P28248 | DCD_ECOLI | Deoxycytidine triphosphate deaminase (EC 3.5.4.13) | dcd | 193 | 0 | 0 |
| P26646 | ACUI_ECOLI | Probable acrylyl-CoA reductase AcuI  (EC 1.3.1.84) | acuI | 324 | 0 | 0 |
| P51981 | AEEP_ECOLI | L-Ala-D/L-Glu epimerase (EC 5.1.1.20) | ycjG | 321 | 0 | 0 |
| P31441 | ADEC_ECOLI | Adenine deaminase (EC 3.5.4.2) | ade | 588 | 0 | 0 |
| P42907 | AGAS_ECOLI | Putative tagatose-6-phosphate ketose/  aldose isomerase (EC 5.3.1.-) | agaS | 384 | 0 | 8 |
| P51020 | HOA_ECOLI | 4-hydroxy-2-oxovalerate aldolase  (EC 4.1.3.39) | mhpE | 337 | 0 | 0 |
| P33898 | G3P2_ECOLI | Putative glyceraldehyde-3-phosphate dehydrogenase C (EC 1.2.1.12) | gapC | 333 | 12 | 44 |
| P07639 | AROB_ECOLI | 3-dehydroquinate synthase (EC 4.2.3.4) | aroB | 362 | 2 | 2 |
| P39451 | ADHP_ECOLI | Alcohol dehydrogenase, propanol-preferring (EC 1.1.1.1) | adhP | 336 | 1 | 8 |
| P75747 | ABRB_ECOLI | Protein AbrB | abrB | 348 | 0 | 0 |
| P19926 | AGP_ECOLI | Glucose-1-phosphatase (EC 3.1.3.10) | agp | 413 | 0 | 0 |
| P07821 | FHUC_ECOLI | Ferric hydroxamate uptake protein C  (EC 3.6.3.34) | fhuC | 265 | 0 | 7 |
| P0ACN7 | CYTR_ECOLI | HTH-type transcriptional repressor CytR | cytR | 341 | 0 | 0 |
| P0AA78 | EXUT_ECOLI | Hexuronate transporter | exuT | 432 | 0 | 0 |
| P0AE14 | AMPE_ECOLI | Protein AmpE | ampE | 284 | 0 | 0 |
| P0AEB0 | CYSW_ECOLI | Sulfate transport system permease protein CysW | cysW | 291 | 1 | 0 |
| P76197 | YDIM_ECOLI | Inner membrane transport protein YdiM | ydiM | 404 | 0 | 0 |
| P0AEE5 | DGAL_ECOLI | D-galactose-binding periplasmic protein | mglB | 332 | 0 | 0 |
| P18335 | ARGD_ECOLI | Succinyldiaminopimelate transferase  (EC 2.6.1.11) | argD | 406 | 0 | 0 |
| P0A6E4 | ASSY_ECOLI | Argininosuccinate synthase (EC 6.3.4.5) | argG | 447 | 0 | 0 |
| P0AEE1 | DCRB_ECOLI | Protein DcrB | dcrB | 185 | 0 | 0 |
| P77379 | RCLR_ECOLI | RCS-specific HTH-type transcriptional activator RclR | rclR | 284 | 0 | 0 |
| P0ABJ1 | CYOA_ECOLI | Cytochrome bo(3) ubiquinol oxidase subunit 2 | cyoA | 315 | 0 | 0 |
| P0ACD8 | MBHL_ECOLI | Hydrogenase-1 large chain (EC 1.12.99.6) | hyaB | 597 | 0 | 0 |
| P31572 | CAIB_ECOLI | L-carnitine CoA-transferase (EC 2.8.3.21) | caiB | 405 | 0 | 0 |
| P77671 | ALLB_ECOLI | Allantoinase (EC 3.5.2.5) | allB | 453 | 1 | 3 |
| P77231 | CITG_ECOLI | 2-(5''-triphosphoribosyl)-3'-dephosphocoenzyme-A synthase  (EC 2.4.2.52) | citG | 292 | 0 | 2 |
| P75925 | C56I_ECOLI | Cytochrome b561 homolog 2 | yceJ | 188 | 0 | 0 |
| P30859 | ARTI_ECOLI | Putative ABC transporter arginine-binding protein 2 | artI | 243 | 1 | 0 |
| P37306 | ARCC_ECOLI | Carbamate kinase (EC 2.7.2.2) | arcC | 297 | 4 | 15 |
| P17583 | CYNX_ECOLI | Cyanate transport protein CynX | cynX | 384 | 0 | 0 |
| P11988 | BGLB_ECOLI | 6-phospho-beta-glucosidase BglB  (EC 3.2.1.86) | bglB | 470 | 0 | 2 |
| P0AAB6 | GALF_ECOLI | UTP--glucose-1-phosphate uridylyltransferase (EC 2.7.7.9) | galF | 297 | 6 | 9 |
| P76052 | ABGB_ECOLI | p-aminobenzoyl-glutamate hydrolase subunit B (EC 3.5.1.-) | abgB | 481 | 0 | 0 |
| P42601 | ALX_ECOLI | Inner membrane protein alx | alx | 321 | 0 | 0 |
| P0AE30 | ARTM_ECOLI | Arginine ABC transporter permease protein ArtM | artM | 222 | 3 | 0 |
| P76460 | ATOE_ECOLI | Short-chain fatty acids transporter | atoE | 440 | 0 | 0 |
| P23890 | CADC_ECOLI | Transcriptional activator CadC | cadC | 512 | 0 | 0 |
| P00509 | AAT_ECOLI | Aspartate aminotransferase (AspAT)  (EC 2.6.1.1) | aspC | 396 | 1 | 1 |
| P09551 | ARGT_ECOLI | Lysine/arginine/ornithine-binding periplasmic protein | argT | 260 | 1 | 2 |
| P52599 | EMRK_ECOLI | Probable multidrug resistance protein EmrK | emrK | 387 | 0 | 0 |
| P0A6C5 | ARGA_ECOLI | Amino-acid acetyltransferase (EC 2.3.1.1) | argA | 443 | 0 | 0 |
| P76472 | ARND_ECOLI | Probable 4-deoxy-4-formamido-L-arabinose-phosphoundecaprenol deformylase ArnD (EC 3.5.1.n3) | arnD | 296 | 0 | 0 |
| P37686 | ADH2_ECOLI | Probable alcohol dehydrogenase  (EC 1.1.1.1) | yiaY | 383 | 1 | 3 |
| P00811 | AMPC_ECOLI | Beta-lactamase (EC 3.5.2.6) | ampC | 377 | 1 | 0 |
| P05804 | BGLR_ECOLI | Beta-glucuronidase (GUS) (EC 3.2.1.31) | uidA | 603 | 0 | 0 |
| P17411 | CHBF_ECOLI | 6-phospho-beta-glucosidase (EC 3.2.1.86) | chbF | 450 | 2 | 1 |
| P06616 | ERA_ECOLI | GTPase Era | era | 301 | 13 | 39 |
| P0A7H0 | RECF_ECOLI | DNA replication and repair protein RecF | recF | 357 | 3 | 2 |
| P23874 | HIPA_ECOLI | Serine/threonine-protein kinase HipA (EC 2.7.11.1) | hipA | 440 | 0 | 0 |
| P37666 | GHRB_ECOLI | Glyoxylate/hydroxypyruvate reductase B (EC 1.1.1.79) | ghrB | 324 | 0 | 2 |
| P0A9S5 | GLDA_ECOLI | Glycerol dehydrogenase (GDH) (GLDH) (EC 1.1.1.6) | gldA | 367 | 0 | 1 |
| P0ABG1 | CDSA_ECOLI | Phosphatidate cytidylyltransferase  (EC 2.7.7.41) | cdsA | 285 | 0 | 1 |
| P0AB91 | AROG_ECOLI | Phospho-2-dehydro-3-deoxyheptonate aldolase, Phe-sensitive (EC 2.5.1.54) | aroG | 350 | 0 | 0 |
| P00888 | AROF_ECOLI | Phospho-2-dehydro-3-deoxyheptonate aldolase, Tyr-sensitive (EC 2.5.1.54) | aroF | 356 | 0 | 0 |
