# Supplementary material for: Comparative genomic analysis of mollicutes with and without a chaperonin system
Source: PLoS One. 2018 Feb 13;13(2):e0192619. doi: 10.1371/journal.pone.0192619 (PMC5810989; doi:10.1371/journal.pone.0192619)
Supplement: S5 Table — (DOCX) [file pone.0192619.s005.docx]

S5 Table. Analysis of codon usage and codon usage entropies

| Clients | | | | | | Non Clients | | | |
| --- | --- | --- | --- | --- | --- | --- | --- | --- | --- |
|  |  |  |  |  |  |  |  |  |  |
| **AA** | **Codon** | **Fraction GroE^-^** | **Fraction GroE^+^** | **Enropy GroE^-^** | **Entropy  GroE^+^** | **Fraction GroE^-^** | **Fraction GroE^+^** | **Enropy GroE^-^** | **Entropy  GroE^+^** |
| A | GCA | 0.428 | 0.419 | 0.482 | 0.501 | 0.421 | 0.446 | 1.564 | 1.665 |
|  | GCC | 0.091 | 0.089 |  |  | 0.08 | 0.1 |  |  |
|  | GCG | 0.058 | 0.084 |  |  | 0.054 | 0.076 |  |  |
|  | GCT | 0.423 | 0.407 |  |  | 0.444 | 0.378 |  |  |
| C | TGC | 0.215 | 0.183 | 0.226 | 0.207 | 0.201 | 0.179 | 0.724 | 0.678 |
|  | TGT | 0.785 | 0.817 |  |  | 0.799 | 0.821 |  |  |
| D | GAC | 0.152 | 0.204 | 0.185 | 0.220 | 0.223 | 0.226 | 0.766 | 0.771 |
|  | GAT | 0.848 | 0.796 |  |  | 0.777 | 0.774 |  |  |
| E | GAA | 0.854 | 0.856 | 0.181 | 0.179 | 0.879 | 0.878 | 0.532 | 0.535 |
|  | GAG | 0.146 | 0.144 |  |  | 0.121 | 0.122 |  |  |
| F | TTC | 0.137 | 0.148 | 0.173 | 0.182 | 0.172 | 0.157 | 0.662 | 0.627 |
|  | TTT | 0.863 | 0.852 |  |  | 0.828 | 0.843 |  |  |
| G | GGA | 0.414 | 0.271 | 0.508 | 0.514 | 0.443 | 0.349 | 1.641 | 1.782 |
|  | GGC | 0.088 | 0.119 |  |  | 0.078 | 0.111 |  |  |
|  | GGG | 0.095 | 0.101 |  |  | 0.086 | 0.124 |  |  |
|  | GGT | 0.403 | 0.509 |  |  | 0.393 | 0.416 |  |  |
| H | CAC | 0.254 | 0.321 | 0.246 | 0.273 | 0.349 | 0.382 | 0.933 | 0.959 |
|  | CAT | 0.746 | 0.679 |  |  | 0.651 | 0.618 |  |  |
| I | ATA | 0.23 | 0.18 | 0.366 | 0.393 | 0.186 | 0.186 | 1.227 | 1.353 |
|  | ATC | 0.103 | 0.181 |  |  | 0.138 | 0.203 |  |  |
|  | ATT | 0.668 | 0.639 |  |  | 0.677 | 0.611 |  |  |
| K | AAA | 0.851 | 0.826 | 0.183 | 0.201 | 0.868 | 0.821 | 0.563 | 0.678 |
|  | AAG | 0.149 | 0.174 |  |  | 0.132 | 0.179 |  |  |
| L | CTA | 0.111 | 0.125 | 0.529 | 0.537 | 0.099 | 0.109 | 1.670 | 1.817 |
|  | CTC | 0.016 | 0.024 |  |  | 0.017 | 0.034 |  |  |
|  | CTG | 0.017 | 0.027 |  |  | 0.016 | 0.022 |  |  |
|  | CTT | 0.161 | 0.138 |  |  | 0.15 | 0.137 |  |  |
|  | TTA | 0.592 | 0.601 |  |  | 0.628 | 0.593 |  |  |
|  | TTG | 0.103 | 0.086 |  |  | 0.09 | 0.105 |  |  |
| M | ATG | 1 | 1 |  |  | 1 | 1 |  |  |
| N | AAC | 0.197 | 0.292 | 0.216 | 0.262 | 0.26 | 0.32 | 0.827 | 0.904 |
|  | AAT | 0.803 | 0.708 |  |  | 0.74 | 0.68 |  |  |
| P | CCA | 0.498 | 0.465 | 0.468 | 0.480 | 0.532 | 0.496 | 1.445 | 1.560 |
|  | CCC | 0.072 | 0.081 |  |  | 0.064 | 0.088 |  |  |
|  | CCG | 0.062 | 0.065 |  |  | 0.037 | 0.051 |  |  |
|  | CCT | 0.367 | 0.39 |  |  | 0.367 | 0.366 |  |  |
| Q | CAA | 0.9 | 0.898 | 0.141 | 0.143 | 0.913 | 0.908 | 0.426 | 0.443 |
|  | CAG | 0.1 | 0.102 |  |  | 0.087 | 0.092 |  |  |
|  |  |  |  |  |  |  |  |  |  |
|  |  |  |  |  |  |  |  |  |  |
|  |  |  |  |  |  |  |  |  |  |
| **AA** | **Codon** | **Fraction GroE^-^** | **Fraction GroE^+^** | **Enropy GroE^-^** | **Entropy  GroE^+^** | **Fraction GroE^-^** | **Fraction GroE^+^** | **Enropy GroE^-^** | **Entropy  GroE^+^** |
| R | AGA | 0.622 | 0.568 | 0.518 | 0.558 | 0.607 | 0.515 | 1.604 | 1.872 |
|  | AGG | 0.07 | 0.05 |  |  | 0.034 | 0.054 |  |  |
|  | CGA | 0.067 | 0.062 |  |  | 0.04 | 0.052 |  |  |
|  | CGC | 0.045 | 0.089 |  |  | 0.051 | 0.085 |  |  |
|  | CGG | 0.023 | 0.03 |  |  | 0.016 | 0.021 |  |  |
|  | CGT | 0.173 | 0.2 |  |  | 0.252 | 0.272 |  |  |
| S | AGC | 0.076 | 0.078 | 0.651 | 0.678 | 0.071 | 0.085 | 2.063 | 2.204 |
|  | AGT | 0.267 | 0.275 |  |  | 0.211 | 0.261 |  |  |
|  | TCA | 0.346 | 0.295 |  |  | 0.444 | 0.354 |  |  |
|  | TCC | 0.041 | 0.058 |  |  | 0.037 | 0.054 |  |  |
|  | TCG | 0.032 | 0.042 |  |  | 0.03 | 0.033 |  |  |
|  | TCT | 0.239 | 0.253 |  |  | 0.207 | 0.214 |  |  |
| T | ACA | 0.463 | 0.397 | 0.456 | 0.518 | 0.527 | 0.447 | 1.499 | 1.644 |
|  | ACC | 0.085 | 0.13 |  |  | 0.092 | 0.118 |  |  |
|  | ACG | 0.036 | 0.075 |  |  | 0.034 | 0.055 |  |  |
|  | ACT | 0.415 | 0.398 |  |  | 0.347 | 0.38 |  |  |
| V | GTA | 0.277 | 0.281 | 0.451 | 0.510 | 0.262 | 0.303 | 1.407 | 1.662 |
|  | GTC | 0.062 | 0.094 |  |  | 0.041 | 0.084 |  |  |
|  | GTG | 0.077 | 0.117 |  |  | 0.077 | 0.106 |  |  |
|  | GTT | 0.584 | 0.508 |  |  | 0.621 | 0.508 |  |  |
| W | TGG | 1 | 1 |  |  | 1 | 1 |  |  |
| Y | TAC | 0.18 | 0.25 | 0.205 | 0.244 | 0.249 | 0.274 | 0.810 | 0.847 |
|  | TAT | 0.82 | 0.75 |  |  | 0.751 | 0.726 |  |  |
| * | TAA | 0.257 | 0.407 | 0.347 | 0.388 | 0.306 | 0.528 | 1.207 | 1.202 |
|  | TAG | 0.069 | 0.071 |  |  | 0.067 | 0.042 |  |  |
|  | TGA | 0.675 | 0.522 |  |  | 0.626 | 0.431 |  |  |
