# Supplementary material for: Comparative genomic analysis of mollicutes with and without a chaperonin system
Source: PLoS One. 2018 Feb 13;13(2):e0192619. doi: 10.1371/journal.pone.0192619 (PMC5810989; doi:10.1371/journal.pone.0192619)
Supplement: S2 Fig — The upper matrix displays substitution events to homologs found in GroE+ mollicutes. The lower matrix shows substitutions in homologs found in GroE- species. (DOCX) [file pone.0192619.s007.docx]

S2 Fig. Amino acid substitution events from *E. coli* non-clients to their homologs [%].

The upper matrix displays substitution events to homologs found in GroE^+^ mollicutes. The lower matrix shows substitutions in homologs found in GroE^-^ species.
