# Supplementary material for: Comparative genomic analysis of mollicutes with and without a chaperonin system
Source: PLoS One. 2018 Feb 13;13(2):e0192619. doi: 10.1371/journal.pone.0192619 (PMC5810989; doi:10.1371/journal.pone.0192619)
Supplement: S3 Fig — The diamonds and squares show the two major clades that contain the GroE system. The triangle marks the species where lateral gene transfer (LGT) was suggested before and circles mark the two additional species for which we suggest LGT events. (DOCX) [file pone.0192619.s008.docx]

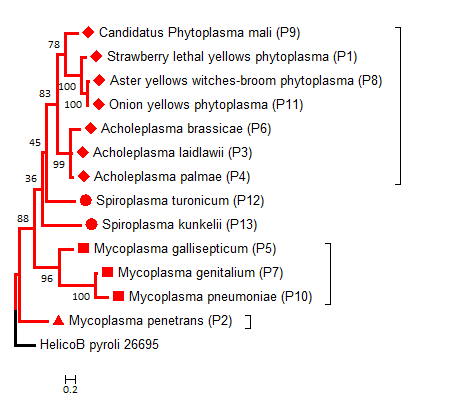

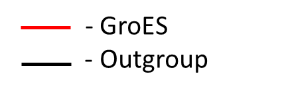


**S3 Fig. Evolutionary tree of GroES sequences of GroE^+^ mollicutes.** The diamonds and squares show the two major clades that contain the GroE system. The triangle marks the species where lateral gene transfer (LGT) was suggested before and circles mark the two additional species for which we suggest LGT events.
